# Clinical evaluation of ophthalmic findings in active, amateur, adult, competitive male boxers in India

**DOI:** 10.22336/rjo.2024.06

**Published:** 2024

**Authors:** Mandhoof Moosa, Jaya Kaushik, Ankita Singh

**Affiliations:** * Department of Ophthalmology, AFMC, Pune, India; ** Department of Ophthalmology, CH Lucknow, Uttar Pradesh, India; *** Department of Ophthalmology, Military Hospital Bathinda, Punjab, India

**Keywords:** boxer, ocular findings, eye injuries

## Abstract

**Background:** In the popular fighting sport of boxing, opponents strike each other above the belt line in the face, chest, and belly. The physical parts most exposed are therefore the nose and eyes. In amateur boxing, fights go only three rounds - three minutes for men and one minute for women - with a one-minute break in between. They wear gloves, but the head protection used in the men’s game has been removed by AIBA due to the high likelihood of concussion when using head protection. Because chronic ocular changes may take longer than the expected short-term effects, this study included at least 3 years of competitive sports participation.

**Study design and setting:** Institutional-based cross-sectional study.

**Materials and methods:** To evaluate ophthalmic outcomes, 200 eyes of 100 active amateur, adult, and competitive male boxers were studied.

**Results:** Of the 100 boxers, 51 had ophthalmic changes in at least one eye, and 49 had normal eyes. The average age of boxers was 24.98 years. The average duration of boxing training was 7.04 years. Healed eyelid scars, subconjunctival hemorrhages, conjunctival papillae, traumatic mydriasis, posterior synechiae, angulation abnormalities, traumatic cataracts, lens subluxation, increased intraocular pressure, and peripapillary atrophy were observed on the ocular side. None of these could be attributed to boxing.

**Conclusion:** Boxing-related eye injuries are common in India and the most common vision-threatening eye abnormalities include traumatic cataracts, lens subluxation, and angle abnormalities. Surprisingly, no macular lesions were found on physical examination and OCT. Additional studies with a larger number of boxers will be needed to evaluate and prevent clinical symptoms. All boxers should have a complete eye exam regularly.

**Abbreviations:** AIBA = Association Internationale de Boxe Amateur, OCT = Optical Coherence Tomography

## Introduction

The incidence of eye injuries due to sporting activities has shown a steady increase in recent times. In 1923, Garrow found that sport accounted for only 0.7% of eye injuries admitted to Glasgow Royal Infirmary [**1**]. By the 1970s, 4.1% was reported from Belfast [**2**]. In 1988, a study from Manchester Royal Eye Hospital reported that sports injuries account for 25.1% of all admissions due to trauma [**3**]. A study from Glasgow in 1989 reported that 42.2% of ocular injuries requiring hospital admission were sports-related [**4**].

In the popular combat sport of boxing, fighters strike each other above the belt line in the face, chest, and belly. Thus, the physical parts most exposed are the nose and eyes. The distance of the strike, the force and placement of the fist, and the usage of protective gear are only a few of the variables that affect eye damage in boxing [**5**]. In amateur boxing, fights go only three rounds - three minutes for men and one minute for women - with a one-minute break in between. Although they wear gloves, the AIBA banned head protection from men’s competition because it increased the risk of concussions.

Boxing is regarded as a high-risk activity for ocular injuries because, like a few other sports, it is inherently traumatizing. The distance of the strike, the force and placement of the fist, and the usage of protective gear are only a few of the variables that affect eye damage in boxing. Between 41% and 76% of boxers have eye injury indicators on their bodies [**6**]. All of the eye’s structures may be affected by these wounds, which may cause irreversible blindness.

Boxers lack systematic long-term follow-up, making it difficult to assess the long-term morbidity of ocular injuries. Giovinazzo VJ et al. [**7**] reported that at least one eye injury occurred in 66% of boxers. Vision-threatening injuries, defined as significant damage to the angle, lens, macula, or peripheral retina, occurred in 58% of boxers. 19% of boxers had angular abnormalities and another 19% had pathological cataracts, of which more than 70% were posterior subcapsular cataracts. Six boxers had macular lesions. 24% of boxers experienced retinal detachment. A significant correlation was found between the total number of attacks, the total number of losses, and the presence or absence of retinal detachment.

Given that post-trauma ocular tissue can show alterations, it can lead to substantial increases in intraocular pressure, being assumed that optic nerve injury will ensue if the pressure is elevated for an extended period [**8**].

In 1955, Doggart suggested that ruptures of Descemet’s membrane, hyphaema, glaucoma, iridodialysis, cataracts, retinal detachments, vitreous hemorrhage, maculopathies, and choroidal ruptures were potential eye injuries sustained during boxing, but reported no data. 

It is frequently reported that participating in this activity might lead to complications [**9**,**10**]. Between 41% and 76% of boxers exhibit symptoms of eye injury [**7**,**11**]. Traditionally, eye injury from boxing has been explained by three mechanisms: equatorial enlargement, indirect coup, and direct coup [**12**]. But, Vinger and Capão Filipe have identified a fourth mechanism called the “suction effect” [**13**]. 

Our study aimed to evaluate the following: visual acuity, intraocular pressure, gonioscopy results, anterior segment, and retinal findings in Indian boxers who have been actively participating in amateur boxing for the past three years. Additionally, we studied the results of Optical Coherence Tomography (OCT) of the macula and optic disc in a subset of boxers. To the best of our knowledge, no research has previously identified ocular complications associated with boxing in India.

## Methods

This cross-sectional study was performed at a tertiary care center, in Pune from August 2020 to January 2022. The study population included young (18 to 35 years), active, amateur, male boxers with a minimum of 03 years in competitive sports with prior visual acuity. The boxers with previous ocular diseases, boxers with eye diseases in the family, and boxers who took anabolic steroids/any other substances banned by the World Anti-Doping Agency (WADA) were excluded from this study. The sample size was calculated to estimate a 95% confidence interval for the proportion of boxers having eye injuries with a 3% absolute error of margin with finite correction (N=110). The sample size worked out to be 100, assuming that the prevalence of eye injury is 51% [**14**]. Socio-professional characteristics like age, gender, number of fights, years of boxing practice, boxers’ performance, and frequency of training were acquired. After giving their written informed consent, every participant had a thorough ophthalmologic examination.

## Results

This cross-sectional study included 100 active, amateur, adult, and competitive male boxers from India. The ocular findings were statistically evaluated using the chi-square test. The statistical analysis was performed on SPSS statistical software. A P-value less than 0.05 was considered statistically significant.

**Fig. 1:**
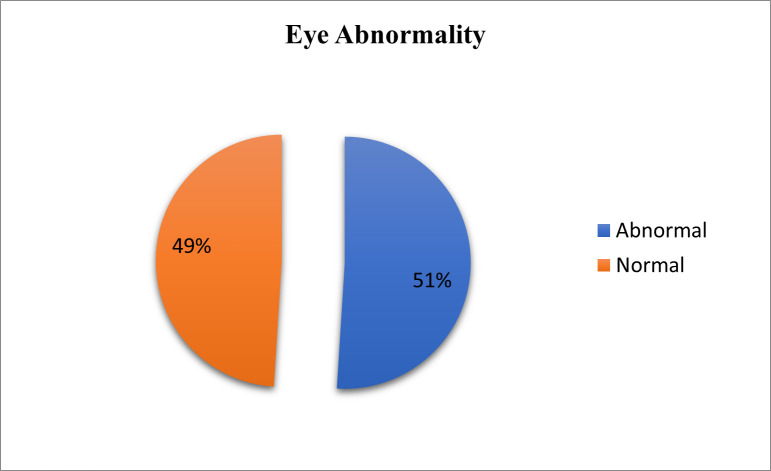
Eye abnormalities in boxers

**Table 1** shows the age group distribution of boxers. Arbitrarily, boxers were grouped into ages between 18 to ≤ 25 years and ages between > 25 years to 35 years as the inclusion criteria included ages between 18 to 35 years. Out of 100 boxers who participated, 51 (51%) boxers were aged between 18 years to ≤ 25 years, and 49 (49%) boxers were aged between > 25 years to 35 years. The youngest boxer was 18 years old and the eldest boxer was 29 years old. **Table 2** summarizes the distribution of amateur boxers concerning years of boxing where, out of 100 boxers, 17 boxers (70%) had 3 to 5 years of boxing experience, 40 boxers (40%) had 5.01 to 7 years of boxing experience, 42 boxers (42%) had 7.01 to 10 years of boxing experience and 1 boxer (1%) had more than 10 years of experience in boxing. **Table 3** shows the distribution of the number of fights in boxers, where 64 boxers (64%) had ≤ 100 fights in their careers, 17 boxers (17%) had 101 to 200 fights in their careers and 19 boxers (19%) had > 200 fights in their careers. **Table 4** and **Fig. 1** show the distribution of overall abnormalities in boxers where 51 out of 100 boxers (51%) had ocular findings and 49 (49%) out of 100 boxers’ eyes were normal.

**Table 1:** Age group distribution of boxers

Age group	Number of boxers	Percentage (%)
18 to ≤ 25	51	51.0
> 25 to 35	49	49.0
Total	100	100.0

**Table 2:** Distribution of boxers concerning years of boxing

Boxing years	Number of boxers	Percentage (%)
03 to 05	17	17.0
5.01 to 07	40	40.0
7.01 to 10	42	42.0
> 10	1	1.0
Total	100	100.0

**Table 3:** Distribution of the number of fights in boxers

Number of fights	Number of boxers	Percentage (%)
≤ 100	64	64.0
101-200	17	17.0
> 200	19	19.0
Total	100	100.0

**Table 4:** Distribution of overall abnormalities in boxers

Overall Abnormality	Number of boxers	Percentage (%)
Abnormal	51	51.0
Normal	49	49.0
Total	100	100.0

**Fig. 2:**
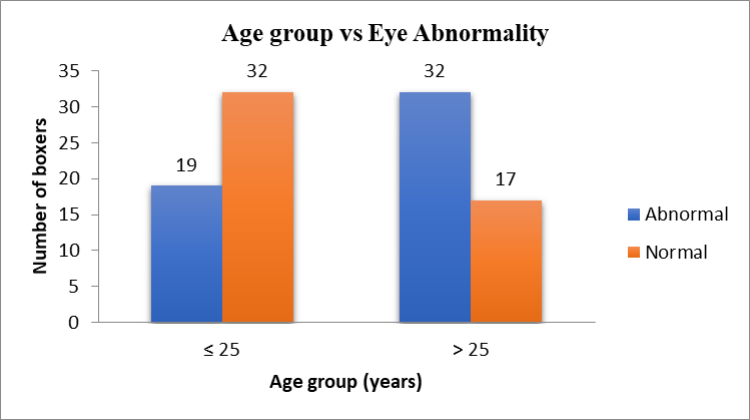
Age group vs. eye abnormality in boxers

**Fig. 3:**
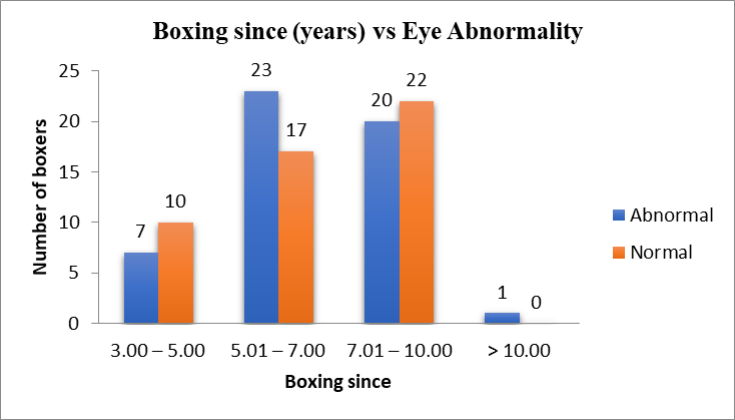
Years of boxing vs. eye abnormality in boxers

**Fig. 4:**
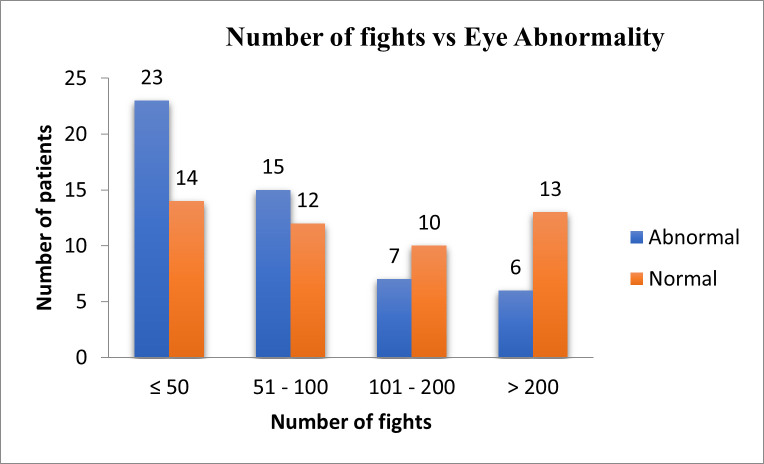
Number of fights vs. eye abnormality in boxers

**Table 5** and **Fig. 2** show the distribution of ocular findings in different age groups in at least one eye, where the p-value was < 0.05 (Significant). The distribution of ocular findings concerning years of boxing is shown in **Table 6** and **Fig. 3** where the p-value > 0.05 (Not Significant) Chi-square test was used. **Table 7** and **Fig. 4** show the distribution of ocular findings concerning the number of fights where the p-value > 0.05 (Not Significant) Chi-square test was used. **Table 8** summarizes the distribution of ocular findings versus the number of eyes.

**Table 5:** Distribution of ocular findings in different age groups in at least one eye

Age group	Abnormality		Total	p-value
	Abnormal	Normal		
18 to ≤ 25	19	32	51	0.006
> 25 to 35	32	17	49	
Total	51	49	100	

**Table 6:** Distribution of ocular findings in years of boxing

Boxing in years	Abnormality		Total	p-value
	Abnormal	Normal		
3.00-5.00	7	10	17	0.478
5.01-7.00	23	17	40	
7.01-10.00	20	22	42	
> 10.00	1	0	1	
Total	51	49	100	

**Table 7:** Distribution of ocular findings concerning the number of fights

Number of fights	Abnormality		Total	p-value
	Abnormal	Normal		
≤ 50	23	14	37	0.140
51-100	15	12	27	
101-200	7	10	17	
> 200	6	13	19	
Total	51	49	100	

**Table 8:** Distribution of ocular findings versus the number of eyes

Ocular findings	Number of boxers	Percentage (%)
Lid and Adnexa	25	25
Conjunctiva	13	13
Iris	7	7
Gonioscopy (angle abnormalities)	4	4
Lens	6	6
Pupillary reflex	5	5
Peripapillary atrophy	2	2
Raised IOP	3	3
Macular lesion	Nil	-

## Discussion

Given the boxers’ high level of motivation and the confidentiality of the data gathered, the research had a 100% participation rate. In contrast to the 28.4 years indicated by Kagmeni et al., the mean age of boxers was 25 years [**14**]. The possible difference may be because Kagmeni et al. included retired boxers.

Arbitrarily, boxers were grouped into ages between 18 to ≤ 25 years and ages between > 25 to 35 years. 51 boxers (51%) were aged between 18 to ≤ 25 years and 49 boxers (49%) were aged between > 25 to 35 years. The mean age of our boxers with ocular findings was 24.98 years and the mean age of boxers without any ocular findings was 23.56 years.

The mean duration of boxing in our boxers was 7.04 years, which was shorter compared to other studies, such as the one by Hazar et al. [**6**] in Turkey and the one by Kagmeni et al. [**14**]. The possible reason for our study might be that we included only boxers aged between 18 to 35 years. Another explanation would be that boxers in affluent nations receive high compensation and guaranteed medical follow-up. They have a lengthy, competitive career as a result.

Years of boxing experience did not significantly correlate with the number of ocular findings in our study (P-value > 0.05 using the Chi-square test). The study’s smaller sample size might be the explanation. 

Also, the association between ocular findings and the number of fights of individuals was not significant (p-value > 0.05 using the Chi-square test). However, we discovered that as the frequency of fights increased, the percentage of ocular findings reduced. It is possible that when a person engages in more fights, they pick up strategies for avoiding most injuries that result from negligence or insufficient expertise.

In our study, ocular findings were found in 51 boxers (51%). Our data was lower than that of Giovinazzo et al. [**7**] and Wedrich et al. [**11**], whose studies reported ocular finding rates among boxers as 66% and 76% respectively. But, Kagmeni et al. [**14**] found 51.42% of boxers with eye injuries, which is similar to our study results. In our study, various ocular changes included healed eyelid scars, subconjunctival hemorrhages, conjunctival papillae, traumatic mydriasis, posterior synechiae, angle abnormalities, traumatic cataracts, lens subluxation, increased intraocular pressure, and peripapillary atrophy. Our study did not include professional boxers, whose fights are much longer and can last up to 12 rounds.

In our study, one hundred boxers (100%) had unaided visual acuity of 6/6. Compared to Kagmeni et al. (2014), who reported visual acuity of 6/6 in 85.71% of cases, this proportion is greater. This may be due to our inclusion criteria of prior normal visual acuity and the exclusion criteria of boxers with previous refractive errors and boxers with eye disease in the family.

We observed that 25% of cases in our research had lid scars. Since there were no recent lid lacerations, the fighters did not need any assistance. Punches to the anterolateral region of the face, the chest, and the belly, above the opponent’s belt line, are used in boxing. Thus, these people tend to have more lid lacerations.

Subconjunctival hemorrhages were found in 12 boxers (12%), which is less than in the study of Kagmeni et al. [**14**] who reported 24.24% cases. The reason for this high incidence was that the research period fell during a time when athletes were training hard to prepare for impending competitions. Bianco et al. [**15**] also found the increased prevalence of subconjunctival hemorrhages in boxers. Conjunctival papillae were found in 1 boxer (1%), which might be due to chronic irritation and frequent trauma to the eye.

In our study, 5 boxers (5%) had traumatic mydriasis, which was less than in the study of Giovinazzo et al. [**7**] who reported 9% of boxers with traumatic mydriasis. This was due to direct blunt trauma to the sphincter muscle of the iris. 7 boxers (7%) had posterior synechiae, which represented less than in the study of Kagmeni et al. [**15**], who reported 12.12% in their series. This might result from elevated intraocular pressure, persistent anterior chamber irritation, or prior uveitis sustained while training.

However, as there was no history and no other findings of uveitis, these posterior synechiae could have been possibly due to chronic irritation or raised intraocular pressure.

The most common sight-threatening problems were found to be lens-related, including traumatic cataracts and subluxated lenses. Three boxers (3%) with traumatic cataracts and three boxers (3%) with subluxated lenses were included in our study. Giovinazzo et al. [**7**] discovered that 19% of boxers in a group of 74 had cataracts. Boxing was an obvious correlation given the patients’ ages and the unilateral nature of these lesions. Since these lesions did not affect their eyesight, the boxers were kept under observation to monitor any decline in their vision and any advancement of cataracts. If necessary, they might then have surgery at a later time. Lens dislocation is typically the result of severe damage to the eye, or minor trauma in those with zonular fragility.

Increased intraocular pressure was seen in 3 boxers (3%) in our study. These boxers were kept in regular follow-up as the intraocular pressure was borderline and no glaucomatous optic nerve or visual field changes were observed. Also, 4 boxers (4%) had angle abnormalities, which included pigment dispersion in 2 boxers and angle recession in 2 boxers, but they did not have any glaucomatous changes like visual field changes, increased intraocular pressure, or optic disc changes. In contrast to the findings of Giovinazzo et al. [**7**] and Wedrich et al. [**11**], who reported angle abnormalities of 19% and 16%, respectively, our study’s findings on angle abnormalities were very modest.

Surprisingly, no macular findings were noted on examination and OCT in our study. In our case series, 13 boxers (13%) were kept in follow-up due to different ocular findings.

This study’s strength was the greater knowledge of ocular problems associated with boxing. This study’s limited sample size and younger age group were its main limitations.

## Conclusion

This study aimed to determine the incidence of eye diseases among currently active adult male amateur boxers in India. Of the 100 boxers we evaluated, 51 (51%) had eye problems. There was no significant correlation between the years of boxing experience and vision test results (p-value > 0.05 according to the Chi-square test). Additionally, no significant association (p-value > 0.05 by Chi-square test) was found between the number of attacks and ophthalmologic outcomes. Examination revealed the following ocular changes: angle abnormalities, traumatic cataracts, lens subluxation, increased intraocular pressure, eyelid scarring, conjunctival papillae, traumatic mydriasis, posterior synostosis, and peripapillary atrophy. Major vision-threatening disorders included traumatic cataracts, lens subluxation, and angle abnormalities. Surprisingly, no macular lesions were found on physical examination and OCT. Thirteen out of 100 boxers (13%) were monitored for various ocular complications related to boxing. Additional studies involving larger samples of boxers will be needed to evaluate and avoid clinical outcomes. It is recommended that all boxers have regular and thorough eye exams.

**Conflict of Interest Statement**

The authors state no conflict of interest.

**Informed Consent and Human and Animal Rights Statement**

Informed consent has been obtained from all individuals included in this study.

**Authorization for the use of human subjects**

Ethical approval: The research related to human use complies with all the relevant national regulations, and institutional policies, as per the tenets of the Helsinki Declaration, and has been approved by the review board at AFMC, Pune on 12 Oct 2017 (IEC/OCT/2017).

**Acknowledgments**

None.

**Sources of Funding**

None.

**Disclosures**

None.
